# The Cationic Cytokine IL-26 Differentially Modulates Virus Infection in Culture

**DOI:** 10.1371/journal.pone.0070281

**Published:** 2013-07-10

**Authors:** Oliver Braum, Michael Klages, Helmut Fickenscher

**Affiliations:** Institute for Infection Medicine, Christian Albrecht University of Kiel and University Medical Center Schleswig-Holstein, Kiel, Germany; University of Regensburg, Germany

## Abstract

Interleukin-26 (IL-26) belongs to the IL-10 cytokine family, is produced by activated T cells, and targets epithelial target cells for signal transduction. Here, we describe the IL-26 effects on the infection of culture cells with recombinant vesicular stomatitis virus (VSV), human cytomegalovirus (HCMV), and herpes simplex virus type 1 (HSV-1) expressing green fluorescent protein. After pre-incubation with recombinant IL-26 and at low multiplicity of infection, VSV showed strongly enhanced infection and replication rates as measured for infectivity, for transcript levels, and for protein expression. Control proteins did not affect VSV infection. The IL-26 effect was independent of the IL-26 receptor and neutralized by anti-IL-26 serum. Pre-incubation of VSV was much more efficient than pre-incubation of the target cells to enhance virus infection. IL-26 increased virus adsorption to target cells as shown by quantitative reverse-transcription PCR. In contrast, the infection of IL-26-treated human fibroblasts with HCMV was inhibited and the infection by HSV-1 was not altered by IL-26. Thus, IL-26 differentially modulates the infection by different enveloped viruses.

## Introduction

The T cellular cytokine interleukin-26 (IL-26) is a member of the IL-10 cytokine family consisting of IL-10, IL-19, IL-20, IL-22, IL-24, IL-26, and the type III interferons (IFN-λ), namely IL-28A, IL-28B, and IL-29. The IL-26 protein shares 24.7% amino acid identity and 47% similarity with IL-10. The IL-10 cytokine family is defined by common properties such as conserved genetic and protein structures and the usage of homologous heterodimeric receptors of the class II cytokine family on target cells. Despite these similarities, the biological effects of the individual cytokines are diverse due to different cellular sources and cell type-specific receptors [1–4].

The *IL26* gene was originally described under the designation AK155 due to its peculiar overexpression in human T cells after growth transformation by the simian γ_2_-herpesvirus or rhadinovirus herpesvirus *Saimiri* [5–10]. The human *IL26* gene is localized between the *IFNG* and *IL22* genes on chromosome 12q15. All three genes are transcribed in the same orientation and show a related expression pattern with partially common regulatory elements [10–14]. IL-26 is expressed by activated T cells and, at lower intensity, also in peripheral mononuclear blood cells, natural killer cells, and macrophage-like synoviocytes from rheumatoid arthritis joints [2,9,10,14–16]. Moreover, IL-26 is a marker for the Th17 subset of CD4+ T helper cells which is characterized by the IL-23-induced transcription of *IL17A*, *IL17F*, *IL22*, IL26, *IFNG*, and *CCL20* [17,18]. *IL26* and *IL22* transcription was also shown for the NKp44^+^ subset of natural killer cells [19,20].

Although many features of the protein structure are conserved among the IL-10 family members, IL-19 and IL-22 seem to act as monomers [21–24] and IL-10 as a homodimer [25,26]. In the case of IL-26, the electrophoretic mobility clearly shifted from denaturing to native conditions, indicating dimer formation of native and recombinant IL-26 [2,9]. In contrast to IL-10 and all other IL-10 family members, the peptide composition of IL-26 has a highly basic predicted isoelectric point (pI) of 10.7 due to several positively charged residues in helix B [2,10,15]. Based on these cationic charges, IL-26 binds strongly to glycosaminoglycans such as heparin, heparan sulphate, and dermatan sulfate on cellular surfaces which act similarly to coreceptors in order to enrich IL-26 on the surface of producer and target cells [15].

In contrast to the promiscuous receptor usage of many other IL-10 family cytokines [27,28], the IL-26 receptor (IL-26R) was identified as the IL-26-specific combination of the long IL-20R1 chain together with the accessory IL-10R2 chain. Binding of IL-26 could be neutralized with specific antibodies against IL-20R1 or IL-10R2 [15,29]. The assembly of the IL-26R complex is probably similar to IL-10R. Here, the cytokine binds first to the cytoplasmic long chain and, after conformational changes of the receptor, the cytokine binds to the accessory short chain [30]. Concerning the biological effects of IL-26, the IL-26R expression on target cells is crucial. IL-20R1 expression is limited to epithelial cell types and not found on hematopoietic cells [10,13,15]. In contrast, the accessory chain IL-10R2 is ubiquitously expressed. IL-20R1 positive tissues are skin, intestine, and lung [13,15,31–33]. Several IL-20R1- and IL-10R2 expressing cell types including the colon carcinoma cell lines Colo-205 and LoVo, primary keratinocytes, and the keratinocyte cell line HaCaT react to IL-26 with the tyrosine phosphorylation of signal transducers and activators of transcription (STAT) 1 and 3 in a dose- and time-dependent manner [15,29,34]. Moreover, IL-26 activates the secretion of IL-8 in LoVo and Colo-205 cells and IL-10 in Colo-205, as well as the surface expression of CD54 in keratinocytes [15].

IL-26 treatment of the colon carcinoma cell line HT-29 slightly decreased cell proliferation and enhanced IL-8 and tumor necrosis factor α secretion and suppressor of cytokine signalling-3 (*SOCS3*) transcription [34]. Additionally, IL-26 activated the mitogen-activated protein (MAP) kinases extracellular-signal-regulated kinase-1/2 (ERK-1/2) and stress-activated protein kinase/c-Jun N-terminal kinase-1/2 (SAPK/JNK), as well as Akt phosphorylation [34]. Experience from different laboratories showed that the IL-26 response of the cell line HT-29 is variable, since forced expression of IL-20R1 was necessary for IL-26-dependent STAT3 phosphorylation in several approaches [1,29,34]. Both, IL-26 and IL-22, act on epithelial cells and induce similar target genes in Colo-205 cells, such as *SOCS3* [19,34].

The current knowledge about the biological role of the T cell-cytokine IL-26 is limited but first hints indicate a possible association with autoimmune diseases. In polymorphic marker studies, the genetic region of *IL26* and *IFNG* was correlated with a gender bias in the susceptibility to multiple sclerosis [35,36]. A common polymorphic marker located 3 kb 3’ of *IL26* gene was significantly associated with rheumatoid arthritis in women but not in men [37]. Additionally, the single nucleotide polymorphism rs2870946 was identified in the *IL26* gene that is associated to ulcerative colitis [38]. As mentioned above, a subset of CD4^+^ T cells, the Th17 lymphocytes, are characterized by the production of IL-17A, IL17F, IL-21, IL-22, and IL-26, and IL-26 mRNA is upregulated during human Th17 differentiation after the induction of the nuclear receptor RORγt in naive cord blood CD4^+^ T cells [39]. In colonic biopsies of patients with inflammatory bowel disease (Crohn’s disease), the number of infiltrating IL-26 positive cells, presumably Th17 cells, was increased, IL-26 mRNA expression was upregulated and correlated with IL-8; IL-22 mRNA expression and IL-26 serum levels were increased [34]. Data from transcription analyses of lesional and non-lesional skin from patients with the autoimmune disease psoriasis showed that Th17-derived cytokines (IL-17A, IL-17F, IL-22 and IL-26) were more abundant than in normal skin [17].

Recent data demonstrate an additional role for IL-26 as a proinflammatory cytokine in rheumatoid arthritis [16,40]. In sera and synovial fluids of rheumatoid arthritis patients, IL-26 concentrations were increased. Fibroblast-like and CD68^+^ macrophage-like synoviocytes were identified as the main cellular sources for IL-26 induced by IL-1β and IL-17A. IL-26 treatment of monocytes in culture induced the production of pro-inflammatory cytokines (IL-1β, IL-6, tumor necrosis factor α) and chemokines such as CCL20. Thus, IL-26 may serve as a promising therapeutic target in rheumatoid arthritis [16].

The family of IL-10 cytokines also includes the IFN-λ proteins IL-28A, IL-28B, and IL-29 which signal through heterodimeric receptor complex of IL-28R1 and IL-10R2 expressed primarily by epithelial cells. The IFN-λs have antiviral, pro-apoptotic, and anti-proliferative properties and are produced by different cell types by the induction of antiviral response [41–45]. This includes the induction of antiviral IFN-stimulated genes (ISGs) such as 2', 5'-*oligoadenylate synthetase*, *Mx1*, and *protein kinase R* in cells that are also induced by type I IFN [46].

Since IL-26R is expressed on epithelial cells, an association with the epithelial defense against infectious pathogens seemed possible at the interface between epithelia and IL-26-producing lymphoid tissue. Therefore, we speculated for antiviral properties of IL-26 and used the rhabdovirus vesicular stomatitis virus (VSV) and the herpesviruses human cytomegalovirus (HCMV) and herpes simplex virus type-1 (HSV-1) as model viruses for infection studies.

## Materials and Methods

### Ethics statement

The primary human fibroblasts were cultivated from dissociated foreskin samples from anonymous donors on the basis of a statement of the Ethikkommission der Medizinischen Fakultät der Christian-Albrechts-Universität zu Kiel, reference number D467/13: “this is to certify that according to the German “Berufsordnung für Ärzte, BO § 15” the use of human fibroblast cultures of foreskin samples does not require the approval of an Ethical Committee, since these foreskin samples originate from indicated surgical procedures and are considered clinical waste. Given the “waste status” of the obtained samples these were collected anonymously”.

### Cell cultures

The cell lines Colo-205 (ATCC CCL-222) [15], Colo-320 (ATCC CCL-220) [15], HT-29 (ATCC HTB-38) [29], and LS411N (ATCC CRL-2159) from human colon carcinoma, A549 (ATCC CCL-185) from alveolar lung adenocarcinoma, HepG2 (ATCC HB-8065) [15] from hepatocarcinoma, MDA-MB 231 (ATCC HTB-26) from mammary carcinoma, MeWo (ATCC HTB-65) from melanoma, PancI (ATCC CRL-1469) [15] from pancreatic carcinoma, HEK 293 (ATCC CRL-1573) and HEK 293T (ATCC CRL-11268) [15] from human embryonic kidney, primary human foreskin fibroblasts, and the *Cercopithecus aethiops* kidney cell line Vero (ATCC CRL-1587) were cultivated in high-glucose Dulbecco’s modified Eagle’s medium (DMEM, PAA, Coelbe, Germany) supplemented with 10% heat-inactivated fetal bovine serum (PAA). Chinese hamster ovary (CHO) cells (ATCC CCL-61) [15] were cultivated with Ham’s F-12 medium supplemented with inactivated 10% fetal bovine serum. The baby hamster kidney cell line BHK-21 (ATCC CCL-10) was cultivated in Eagle’s Minimum Essential Medium (PAA) supplemented with 5% fetal bovine serum. The expression plasmids for the reconstitution of the IL-26 receptor (IL-26R) chains IL-20R1 and IL-10R2 (plasmids pEF2-IL-20R1 and pCEP4-IL-10R2long) were kindly provided by Jean-Christophe Renauld (Brussels, Belgium). HT-29 cells were stably transfected with the PvuI-linearized IL-20R1 expression plasmid by nucleofection (Lonza, Cologne, Germany) according to the manufacturer’s protocol and medium was supplemented with G418 sulphate (1 mg/ml, PAA) for selection. CHO cells were first stably transfected with the PvuI-linearized IL-20R1 expression plasmid and IL-20R1-expressing clones were then transfected with the episomal IL-10R2 plasmid and supplemented with 0.2 µg/ml hygromycin B (PAA) for selection. The IL-20R1 expression was verified by Western blot and immunofluorescence with an anti-IL-20R1 monoclonal antibody generated in our laboratory. As seconddary antibodies anti-mouse IgG conjugated with Alexa-Fluor-488 (Life Technologies, Darmstadt, Germany) and anti-mouse IgG conjugated with horse-raddish peroxidase (DakoCytomation. Hamburg, Germany) were used. IL-26-induced STAT3 activation was analyzed with a Tyr 705-phosphospecific antibody (#9131, Cell Signaling, Frankfurt, Germany) and total STAT3 protein was detected as control (#9132, Cell Signaling) in stably transfected HT-29 and CHO cells. To maintain stable transgene expression in HT-29 and CHO clones, G418 sulphate and/or hygromycin B were included in the cell culture medium at 1.0 and 0.2 mg/ml, respectively. Oligonucleotide primers and probes were synthesized by Biomers (Ulm, Germany) or by TIB MOLBIOL (Berlin, Germany) and used for the verification of IL-20R1 and IL-10R2 transcription after cDNA synthesis (Tab. 1).

**Table 1:** Oligonucleotide primers and probes.

**Target gene**	**Type**	**Purpose**	**Sequence (5’-3’)**	**Reference**
GAPDH	forward	RT-PCR	GCA GGG GGG AGC CAA AAG GG	[15]
GAPDH	reverse	RT-PCR	TGC CAG CCC CAG CGT CAA AG	[15]
GAPDH	forward	qRT-PCR	gAA ggT gAA ggT Cgg AgT C	[52]
GAPDH	reverse	qRT-PCR	gAA gAT ggT gAT ggg ATT TC	[52]
GAPDH	probe	qRT-PCR	6-FAM-CAA gCT TCC CgT TCT CAg CCT-BBQ	[52]
GFP	forward	qRT-PCR	aag tcg tgc tgc ttc atg tg	Own design
GFP	reverse	qRT-PCR	acg taa acg gcc aca agt tc	Own design
GFP	probe	qRT-PCR	6-FAM-ccg tag gtg gca tcg ccc tc-TAMRA	Own design
GST (His)	forward	Cloning	GAT CCC ATC ACC ATC ACC ATC ACT AAC	Own design
GST (His)	reverse	Cloning	TCG AGT TAG TGA TGG TGA TGG TGA TGG	Own design
IL-20R1	forward	RT-PCR	TCA AAC AGA ACG TGG TCC CAG TG	[28]
IL-20R1	reverse	RT-PCR	TCC GAG ATA TTG AGG GTG ATA AAG	[28]
IL-10R2	forward	RT-PCR	TAT TGG ACC CCC TGG AAT	Own design
IL-10R2	reverse	RT-PCR	GTA AAC GCA CCA CAG CAA G	Own design
IRF-1	forward	qRT-PCR	CTA AGA GCA AGG CCA AGA GGA A	Own design
IRF-1	reverse	qRT-PCR	CTG CTG AGT CCA TCA GAG AAG GT	Own design
IRF-1	probe	qRT-PCR	6-FAM-TCA GGG CTG GAA TCC CCA CAT GA-TAMRA	Own design
SOCS-3	forward	RT-PCR	CGC GAA GGC TCC TTT GTG GA	Own design
SOCS-3	reverse	RT-PCR	CAG CTT GCG CAC TGC GTT CA	Own design
SOCS-3	forward	qRT-PCR	CTT TCT GAT CCG CGA CAG CT	[51]
SOCS-3	reverse	qRT-PCR	TCA CAC TGG ATG CGC AGG T	[51]
SOCS-3	probe	qRT-PCR	6-FAM-CCA GCG CCA CTT CTT CAC GCT CAG-TAMRA	[51]

### Protein purification and recombinant proteins

N-terminally His-tagged IL-26 and C-terminally His-tagged glutathione-S-transferase (GST) were bacterially expressed in *E. coli* and purified with nickel-chelate chromatography under denaturing conditions for IL-26 [10,15] and under native conditions for GST. The His tag was cloned at the C-terminal position into the GST expression plasmid pGEx-6p-1 (GE, Freiburg, Germany) after PCR (Tab. 1). The correct insertion was verified by sequencing. Protein expression of GST in BL21-CodonPlus (DE3)-RIL (Stratagene, Amsterdam, The Netherlands) was induced with 1.5 mM isopropyl-β-D-1-thiogalactopyranoside. After 4 h, the bacterial cell pellet was lysed in buffer A (50 mM NaH_2_PO_4_, 300 mM NaCl, 10 mM imidazole, pH 8.0). After treatment with lysozyme (1 mg/ml, Roche, Mannheim, Germany) on ice for 30 min, the bacterial pellet was sonicated and the supernatant was mixed with nickel-nitrilotriacetic acid superflow resin (Qiagen, Hilden, Germany) for 1 h at 4°C. The mixture was packed on a column and washed with buffer B (buffer A with 20 mM imidazole). Fractions were eluted with buffer C (buffer A with 250 mM imidazole). Pooled fractions of GST-protein were dialysed twice against phosphate-buffered saline (pH 8.0), sterile filtered, and frozen with 10% glycerol. Purity and concentration of recombinant GST and IL-26 proteins were determined by denaturing protein gel electrophoresis. The lipopolysaccharide concentration as measured by a colorimetric limulus test (Chromo-LAL, Associates of Cape Cod, Inc., East Falmouth, USA) was approximately 0.01 EU/µg IL-26 protein and, thus, too low for interfering effects.

### Virus cultures

The VSV recombinants VSV-GFP (expressing soluble GFP [47]) and VSV-G/GFP (expressing a glycoprotein-GFP fusion protein [48]) were propagated with a multiplicity of infection (MOI) of 0.1 on approximately 70% confluent BHK-21 cells. After 2-30 h, supernatants were collected and virus titers were quantified in quadruplicates in 96-well plates on BHK-21 or Vero cells as plaque-forming units (pfu) per ml by fluorescence microscopy (approximately 1.25 x10^8^ pfu/ml for VSV-GFP and approximately 3.5 x 10^8^ pfu/ml for VSV-G/GFP). The recombinant GFP-expressing HCMV strain AD169 [49] was propagated and titrated in quadruplicates in primary human fibroblasts in low cell passage numbers. After 8 d, supernatants were pooled and the virus titer was quantified by serial dilution on human fibroblasts (approximately 1x10^6^ pfu/ml). The GFP-expressing HSV-1 recombinant C12 derived from strain SC16 [50] was propagated for 3 d and the virus titer of supernatant was determined on Vero cells (approximately 1x10^6^ pfu/ml) in quadruplicates. Either VSV supernatant or seeded target cells were pre-incubated with recombinant IL-26 or GST for 15-30 min at 37°C in cell culture medium (DMEM) without fetal bovine serum in triplicates. For the neutralization of IL-26 effects on the VSV or HCMV infection, the cytokine was pre-incubated at 1-5 µg/ml or 2.5-25 µg/ml, respectively, in cell culture medium with rabbit polyclonal anti-IL-26- or pre-immune serum (1 or 3% v/v) for 15 min at 37°C. Target cells were seeded in 6- or 24-well plates the day before and subsequently infected with a defined MOI between 0.01 and 0.001. After 12 h, GFP expression was visualized by fluorescence microscopy or the cells were detached with accutase (PAA), centrifuged, and fixed with 2% paraformaldehyde (PFA) in phosphate-buffered saline for flow cytometry analysis. The incubation time after the infection of various cell lines was stopped by fixation at 12-18 h depending on the VSV infection rates of the different target cells. Primary human fibroblasts or HCMV were pre-incubated for 5 min with recombinant IL-26 or IL-2 (Proleukin, Novartis, Nuremberg, Germany). The cells were subsequently infected with HCMV (MOI = 0.003) and GFP expression was monitored by fluorescence microscopy on d 2, 4, and 5 post infection. Alternatively, the fibroblasts were detached with trypsin (Biochrom, Berlin, Germany), fixed with 2% PFA, and GFP-positive cells were quantified by flow cytometry. HSV-1 was pre-incubated with recombinant IL-26 or GST protein and Colo-205 cells were infected (MOI = 0.01). After 2 d, the percentage of GFP-positive cells was quantified by flow cytometry or fluorescence microscopy. For the quantification of virus binding to target cells, VSV-GFP was pre-incubated with recombinant IL-26, GST (1 or 5 µg/ml) or polybrene (0.25 or 0.025 µg/ml) in culture medium for 15 min at room temperature, followed by 10 min on ice. The adsorption of pre-incubated VSV-GFP to Colo-205 cells was done for 20 min (MOI = 0.01) on ice and non-adsorbed VSV-GFP particles were rinsed off twice with ice-cold phosphate-buffered saline.

### Microfluorimetric analysis and flow cytometry

In microtiter plates, fibroblasts or Vero cells were treated in phenol red-free DMEM (Biochrom) with IL-26 (10-40 µg/ml) or control proteins and infected with HCMV or HSV-1, respectively. The fluorescence intensity (relative light units, RLU) was measured at several time points post infection in a Saphire 2 fluorescence reader (Tecan, Crailsheim, Germany). The viability of treated, uninfected fibroblasts and Vero cells was verified by measuring the absorbance of the cell-proliferation reagent WST-1 (Roche) at the end of the experiment. The percentage of fixed GFP-positive cells was quantified by flow cytometry with a FACS Canto instrument (Becton Dickinson, Heidelberg, Germany). Flow cytometry results were analyzed with the FCS express software (De Novo, Los Angeles, CA). The autofluorescence of non-infected, non-treated cells was set to 1% by appropriate marker position.

### Quantitative reverse-transcription polymerase-chain reaction

For the quantification of GFP transcripts and VSV adsorption, total RNA was isolated from Colo-205 cells with Trizol (Life Technologies) according to the manufacturer’s protocol. After treatment with DNase I (Roche) for 30 min at 37°C, the RNA was purified with the RNeasy Mini kit (Qiagen). In the two-step quantitative reverse-transcription PCR (RT-PCR), 5 µg of RNA was used for cDNA synthesis with Superscript II reverse transcriptase (Life Technologies) and Oligo (dT)_18_ Primer (Fermentas, Sankt Leon-Rot, Germany) for GFP transcription or random hexamer primer (Fermentas) for VSV quantification according to the manufacturer’s protocol. The cDNA was diluted 1:10 (GFP transcription) or 1:3 (VSV quantification) with DNase- and RNase-free water and the number of transcripts of the virally encoded *GFP*, cellular *SOCS3* [51], IFN-regulating factor 1 (IRF1), VSV genomes (*GFP*) and glycerol aldehyde phosphate dehydrogenase (*GAPDH*) [52] as endogenous housekeeping gene were quantified using the QuantiTect Probe kit (Qiagen) with a light cycler 1.5 instrument (Roche, for *GFP* transcription) or a 7500 real time PCR system (Life Technologies, for VSV quantification). The oligonucleotide primers and probes used for quantitative RT-PCR are described in Tab. 1. As positive controls, the cDNAs for GAPDH, GFP, SOCS-3, and IRF-1 were cloned with the TOPO TA cloning kit into the pCR2.1-TOPO vector (Life Technologies) with the respective forward and reverse primers und the sequence was confirmed.

### Data Analysis

Data from virus titration, flow cytometry, fluorimetry, and from quantitative RT-PCR were statistically analyzed by the Student’s t test with the Prism 5 software (version 5.04, GraphPad Software, La Jolla, USA). A *P* value of <0.05 was considered significant (symbol *). In the figures, *P* values of <0.01 and <0.001 are indicated by the symbols ** and ***, respectively.

## Results

### IL-26-enhanced VSV infection and replication

The rhabdovirus VSV is a widely used enveloped RNA model virus and capable of infecting a broad spectrum of target cells [53] including IL-26-responsive target-cell types such as Colo-205. Two GFP-expressing VSV recombinants, namely VSV-GFP and VSV-G/GFP, were used for the simple quantification of infected target cells [47,48]. VSV-GFP expresses soluble GFP after infection, whereas a GFP-glycoprotein G fusion protein is produced after infection with VSV-G/GFP. Additionally, recombinant GFP-expressing herpesviruses, namely the HCMV strain AD169 [49] and the HSV-1 strain SC16 [50], were included in the experiments to compare IL-26-dependent effects on different virus species.

**Figure 1:**
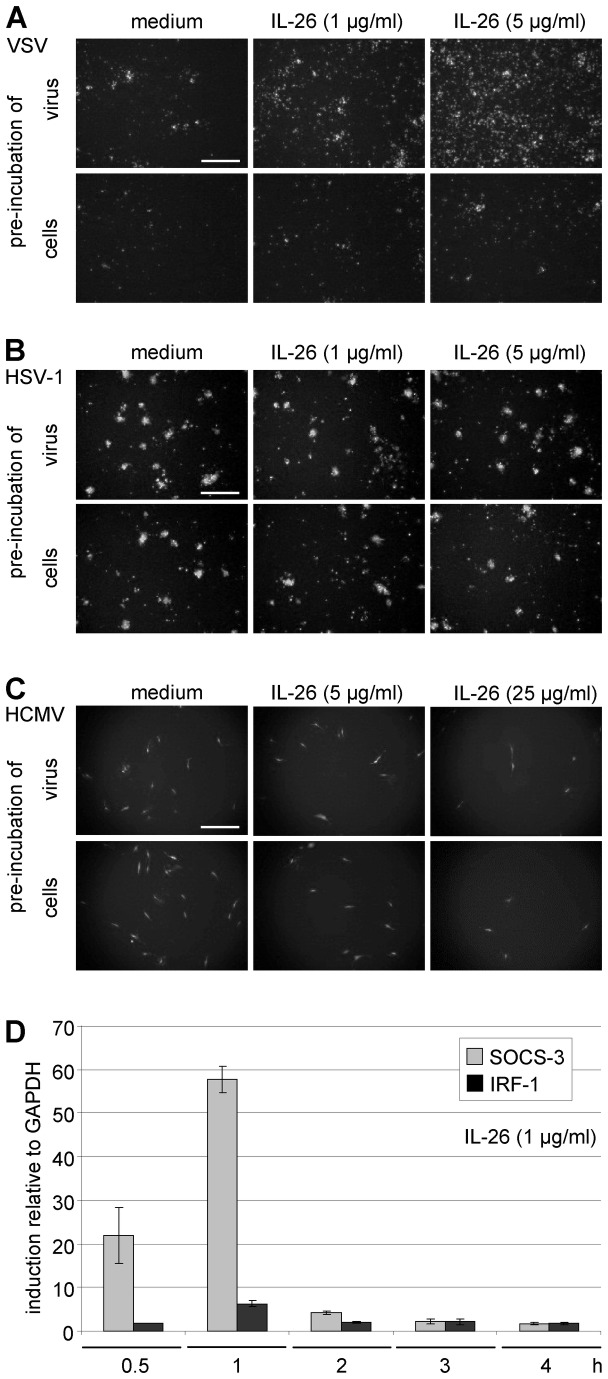
IL-26 effects on infection by VSV, HSV-1, and HCMV. (A, B) VSV-GFP or HSV-1-GFP C-12 (upper panels) or Colo-205 cells (lower panels) were pre-incubated with recombinant IL-26 (1 or 5 µg/ml) or mock-treated for 15 min and infected (MOI = 0.01). After 12 h (VSV) and 2 d (HSV-1), the microscopic images show virus-infected GFP-expressing cells by fluorescence (Olympus IX81, U PLAN FLN, 4x objective, scale bars = 500 µm, representative microscopic fields). (C) HCMV-GFP (strain AD169, upper panels) or primary human fibroblasts (lower panels) were pre-incubated with recombinant IL-26 (5 or 25 µg/ml) for 5 min and infected, (MOI = 0.003). Infected cells were visualized by fluorescence microscopy after 5 d. (D) Colo-205 cells were incubated with recombinant IL-26 (1 µg/ml) or medium for 0.5-4 h. The induction factors for the IL-26-target genes *SOCS3* and *IRF1* relative to the housekeeping gene *GAPDH* were analyzed by quantitative RT-PCR. Mean results with standard deviations from duplicates from two independent experiments are shown.

After pre-incubation of VSV with IL-26 (1 or 5 µg/ml) or medium and subsequent infection of colon carcinoma Colo-205 cells at MOI = 0.01, a strong IL-26 concentration-dependent increase of the number of infected, GFP-expressing cells was observed in relation to medium-treated VSV as shown by fluorescence microscopy after 12 h (Figure 1A, upper panels). In contrast, cell pre-incubation with the cytokine increased only slightly the amount of infected cells (Figure 1A, lower panels). There was no effect of IL-26 treatment on HSV-1 infection in Colo-205 cells after 2 d irrespectively of whether VSV or target cells were pre-incubated (Figure 1B). In contrast to VSV, the infection of HCMV was inhibited by IL-26-pre-incubation (5 or 25 µg/ml) of HCMV or primary human fibroblasts and subsequent infection with HCMV at MOI = 0.003 after 5 d (Figure 1C). Fibroblasts were used for these experiments because HCMV was unable to induce virus-encoded GFP-expression in Colo-205 cells. Primary human fibroblasts are not responsive to IL-26 because they lack both IL-20R1 expression and STAT3 activation after IL-26 stimulation (data not shown). In comparison to VSV, higher IL-26 concentrations (at least 5 µg/ml) were necessary for effects on HCMV infection and the IL-26 pre-incubation of the HCMV supernatant or of the target cells showed similar results. Thus, IL-26 modulates the infection and replication of different virus species differentially.

Target-gene expression was analyzed from Colo-205 cells treated with IL-26 by quantitative RT-PCR in order to demonstrate that functional IL-26 concentrations were used. These target genes were selected due to their highest induction values in a comparative cDNA chip hybridization experiment which was kindly performed by Robert Häsler, Institute for Clinical Molecular Biology, Kiel (GeneChip Human Genome U133 Plus 2.0 Array Affymetrix, Santa Clara, CA). The autoregulatory target gene *SOCS3* was rapidly and strongly induced already within 30 min (up to the induction factor 58 at 60 min) and *IRF1* was rather moderately upregulated (up to factor 6) as normalized on the housekeeping gene *GAPDH* and relative to medium controls (Figure 1D). Within 2 h after IL-26 stimulation, the numbers of SOCS-3 transcripts returned to low basal levels. This corresponded to STAT1 and STAT3 phosphorylation in Colo-205 and other IL-26-responsive cell lines reaching maximal activation within few minutes and after 1 h signalling was already switched off [15].

**Figure 2:**
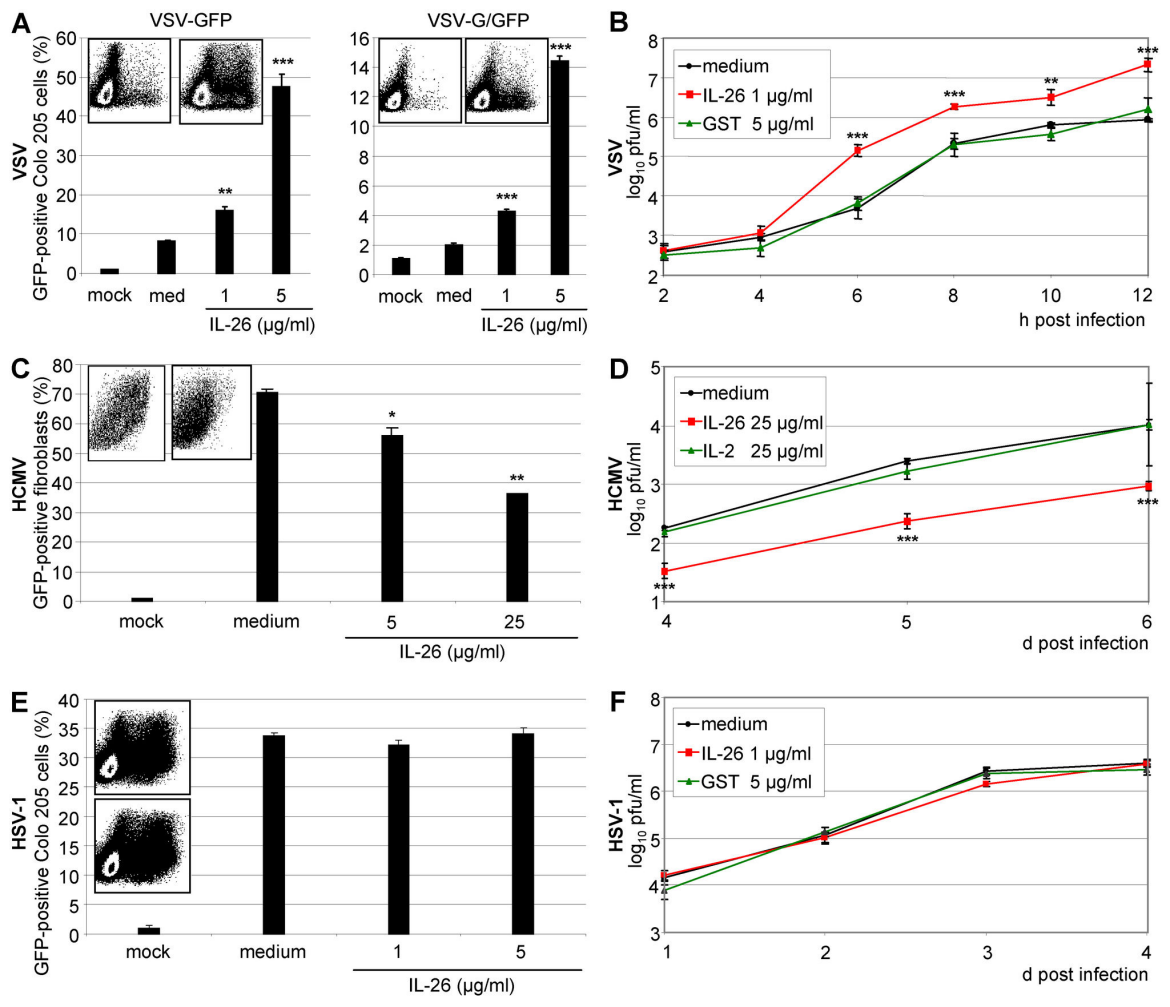
Quantification of the IL-26 effects on the replication of VSV, HSV-1, and HCMV. (A) Recombinant VSV expressing cytoplasmic GFP (VSV-GFP; left) or a GFP-glycoprotein G fusion protein (VSV-G/GFP; right) were pre-incubated with IL-26 (1 or 5 µg/ml) for 15 min and used for the infection of Colo-205 cells (MOI = 0.01). Cells were fixed after 12 h and GFP-positive cells were measured by flow cytometry. The density plots show the GFP fluorescence and the side scatter for medium- (left) and IL-26-treated virus (right, 5 µg/ml). (B) VSV supernatants were pre-incubated with IL-26 (1 µg/ml) or GST (5 µg/ml) for 15 min and used for the infection of Colo-205 cells (MOI = 0.01). At depicted time points, supernatants were titrated on Vero cells. (C) Primary fibroblasts were pre-incubated for 5 min with IL-26 and infected with HCMV-GFP (MOI = 0.003). After 5 d, the percentage of GFP-expressing cells was quantified by flow cytometry. The inserts show density plots with fluorescence and side scatter for medium- (left) and IL-26-treated fibroblasts (right, 25 µg/ml). (D) HCMV titers were determined from d 4-6 post infection by titration on primary fibroblasts after treatment with IL-26, IL-2 (both 25 µg/ml), or medium. (E) HSV-1-GFP supernatant was pre-incubated with IL-26 (1 or 5 µg/ml) for 15 min and Colo-205 cells were infected (MOI = 0.01). After 2 d, the percentage of infected cells was determined by flow cytometry. The inserts show density plots with fluorescence and side scatter for medium- (left) and IL-26-treated fibroblasts (right, 5 µg/ml). (F) HSV-1-GFP titers were determined by titration on Vero cells. Logarithmic mean results with standard deviation of representative experiments are shown for the quantification of virus-infected GFP-expressing cells by flow cytometry and titration (quadruplicates). The symbols *, **, and *** indicate *P* values of <0.05, <0.01, and <0.001, respectively (A, B, C, and D), relative to medium as determined by the Student’s t test.

The strong enhancing effect of IL-26 on VSV infection was quantified by flow cytometry. VSV expressing free cytoplasmic GFP (VSV-GFP) was pre-incubated with IL-26 (1 or 5 µg/ml) in cell-culture medium for 15 min and used for infecting Colo-205 cells (MOI = 0.01). The cells were fixed after 12 h and the number of GFP-expressing cells was measured by flow cytometry (Figure 2A). In comparison to medium-treated VSV, the percentage of infected cells was three- to five-fold increased by recombinant IL-26 in a concentration-dependent manner (Figure 2A, left panel). The numbers of Colo-205 cells expressing the GFP-glycoprotein fusion (VSV-G/GFP) were also enhanced by IL-26 up to five-fold (Figure 2A, right panel). In comparison to VSV-GFP, the percentage of GFP-expressing cells was lower with VSV-G/GFP due to the lower fluorescence signal intensity but the IL-26-dependent enhancement factor was similar to VSV-GFP.

Furthermore, virus replication was measured directly by limiting dilution kinetics. The effect of the recombinant, His-tagged IL-26 was compared with plain medium and with recombinant GST protein as control, lacking the cationic properties of IL-26 but with a similar purification tag and similar molecular size. Therefore, we expressed recombinant GST with C-terminal His tag in *E. coli* with a predicted isoelectric point of 6.7. After nickel-chelate chromatography, recombinant GST protein was used as control protein in parallel to IL-26 in infection experiments. Recombinant IL-26 increased not only the number of VSV-infected cells but also the release of infectious virus particles into the supernatant of infected Colo-205 cells (Figure 2B). VSV supernatant was pre-incubated with IL-26 or GST (1 µg/ml) as control protein and supernatants were collected 2-12 h after the infection of Colo-205 cells (MOI = 0.01). The supernatants were used to infect permissive Vero cells and the virus titer (log_10_ of pfu/ml) was quantified by counting fluorescent cells after serial dilution by fluorescence microscopy and virus growth curves were generated. From 4–12 h post infection, IL-26 increased the virus titers about ten-fold in contrast to medium- or GST-treated virus (Figure 2B).

In contrast to VSV, the percentage of HCMV-infected human fibroblasts was reduced by IL-26 pre-incubation as quantified by flow cytometry after 5 d (Figure 2C). Consequently, the HCMV titers from IL-26-treated fibroblasts were about ten-fold lower than the titers reached with medium or IL-2 as control on d 4, 5, and 6 post infection (Figure 2D). Commercially available IL-2 was used for control since fibroblasts lack the IL-2 receptor. Similarly, the expression of HCMV proteins (IE1, pp65, UL44) was reduced after the IL-26-pre-incubation of fibroblasts as determined by Western blot (data not shown). IL-26 (1 or 5 µg/ml) did not influence HSV-1 infection. Neither frequencies of infected cells nor virus titers were altered on d 2 post infection (MOI = 0.01; Figure 2E, F) confirming the results from fluorescence microscopy. The control proteins GST and IL-2 used for VSV and HCMV infection, respectively, did not influence the resulting virus titers from infected fibroblasts or Colo-205 cells (Figure 2B, D). Moreover, the buffers used for refolding and dialysis after purification of the recombinant proteins did not influence VSV infection rates in Colo-205 cells (data not shown).

**Figure 3:**
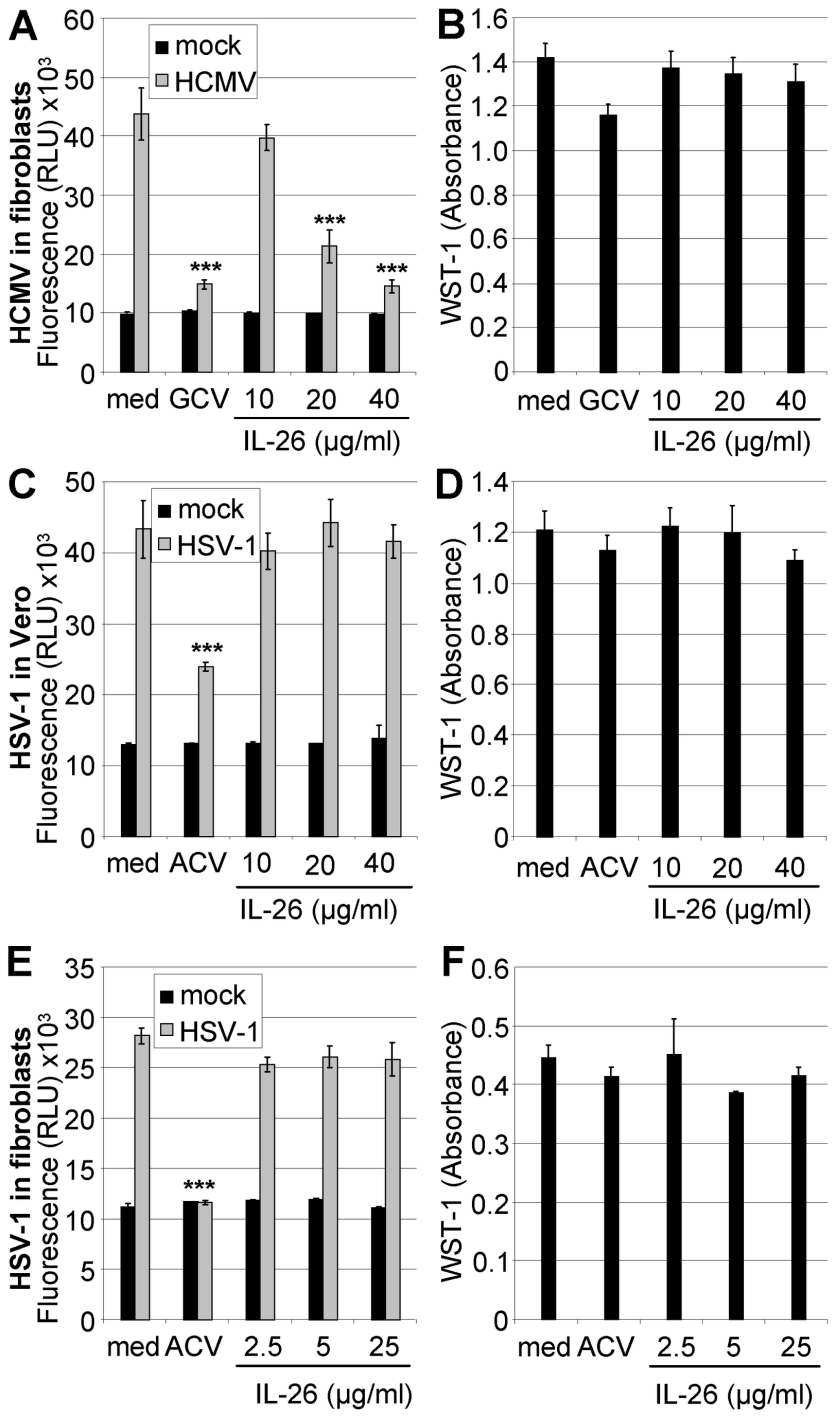
Comparison of IL-26 effects on HCMV and HSV-1 infection. (A) Primary human fibroblasts were pre-incubated with IL-26 (10-40 µg/ml) or GCV (0.05 mM) in phenol-red free medium and infected with HCMV-GFP (MOI = 0.03). After 5 d, fluorescence intensity (RLU) of infected cells was measured with a plate fluorimeter. (C) Vero cells were pre-incubated with IL-26 (10-40 µg/ml) or ACV (0.1 mM) and subsequently infected with HSV-1-GFP (MOI = 0.03). After d 2, the infection was quantified using a plate fluorimeter. (E) Human fibroblasts were pre-incubated with IL-26 (2.5-25 µg/ml) or ACV (0.1 mM) and infected with HSV-1-GFP (MOI = 0.003). After 5 d, RLU values were measured. The viability of IL-26-, ACV-, or GCV-treated uninfected cells was measured by absorbance using the cell-proliferation agent WST-1 on d 6 (B) or d 3 (D) for fibroblasts and on d 2 (F) for Vero cells. Mean data from a representative experiment with standard deviation from quadruplicates are shown. The symbol *** indicates *P* values of <0.001 (A, C and E), relative to medium as determined by the Student’s t test.

The HSV-1 infection of Vero cells and fibroblasts on d 2 and 5 post infection, respectively, was further analyzed using a plate fluorimeter. Whereas the antiviral drug aciclovir (ACV) strongly suppressed virus replication, IL-26 (up to 40 µg/ml) did not alter the fluorescence signals (Figure 3C, E). In a similar setup, ganciclovir (GCV) suppressed HCMV replication in fibroblasts to a similar degree as IL-26 (Figure 3A). The retained viability of IL-26- or drug-treated uninfected cells was verified with a colorimetric test (Figure 3B, D, F).

### Specificity of the IL-26-effect on virus infection and replication

**Figure 4:**
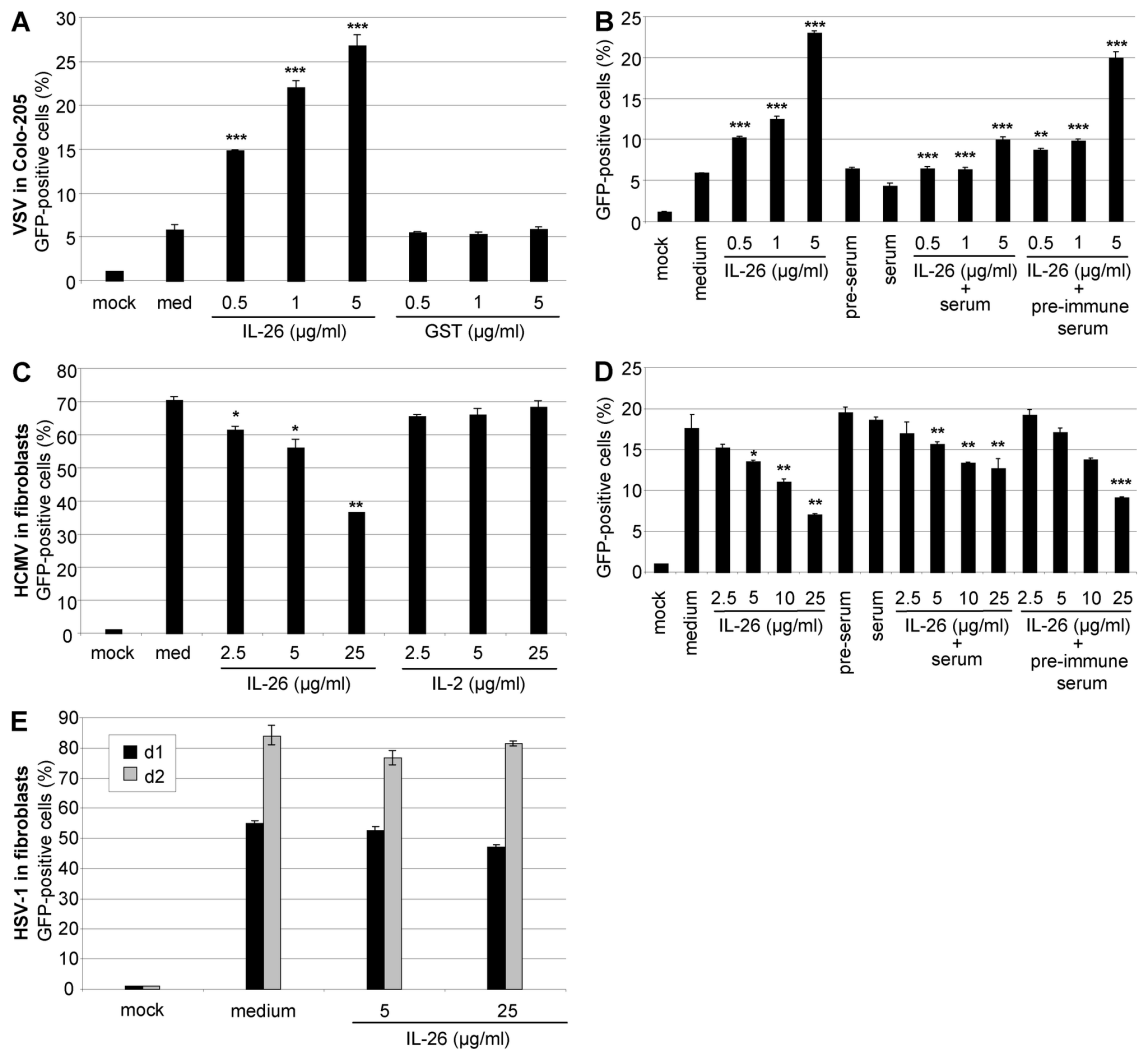
Specificity of IL-26 effects on VSV and HCMV infection. (A) VSV-GFP was pre-incubated with IL-26 or GST (0.5-5 µg/ml) for 15 min and used for the infection of Colo-205 cells (MOI = 0.01). GFP-positive cells were measured by flow cytometry. (B) For the neutralization of IL-26 effects (0.5-5 µg/ml), the protein was pre-incubated with anti-IL-26- or pre-immune serum (1% v/v) at 37°C. After 15 min, VSV-GFP was added and Colo-205 cells were infected (MOI = 0.01). GFP-positive cells were measured by flow cytometry. (C) Fibroblasts were pre-incubated for 5 min with IL-26 or IL-2 (2.5-25 µg/ml) and infected with HCMV-GFP (MOI = 0.003). The percentage of fluorescent fibroblasts was quantified by flow cytometry on d 5. (D) The IL-26-dependent antiviral effect on HCMV was neutralized by pre-incubation of the cytokine (2.5-25 µg/ml) with anti-IL-26 serum 3% (v/v) but not with pre-immune serum. After 15 min, fibroblasts were infected with HCMV (MOI = 0.003). After 4 d, infected fibroblasts were quantified by flow cytometry. (E) HSV-1-GFP C-12 supernatant was pre-incubated with IL-26 (5 or 25 µg/ml) and used for the infection of fibroblasts (MOI = 0.001). On d 1 and 2 post infection, the percentage of infected cells was quantified by flow cytometry. Mean results with standard deviations of a representative experiment are shown. The symbols *, **, and *** indicate *P* values of <0.05, <0.01, and <0.001, respectively (A, B, C, D) as determined by the Student’s t test. The statistical test was performed relatively to the medium-treated infected sample in panels A and C, as well as in B and C (only left part). In panels B and D, the values in presence of serum (central part) were tested in comparison to the samples which were treated with IL-26 alone (left part); the values in presence of pre-immune serum (right part) were tested in comparison to the samples which were treated with IL-26 and immune serum (central part).

The numbers of VSV-infected Colo-205 cells were increased in a dose-dependent manner up to five-fold after pre-treatment with IL-26 in comparison to medium or GST as control as measured by flow cytometry (Figure 4A). A polyclonal anti-IL-26 serum from rabbit was capable of neutralizing IL-26-dependent STAT3 phosphorylation of Colo-205 cells, in contrast to pre-immune sera [15]. The rabbit serum was used for neutralization of the IL-26 effects, in order to demonstrate the IL-26-specificity on VSV and HCMV infection. The cytokine (0.5-5 µg/ml) was mixed with anti-IL-26- or pre-immune serum (1% (v/v)) in culture medium for 15 min at 37°C for antibody binding. Subsequently, VSV was added, Colo-205 cells were infected (MOI = 0.01), and the infected cells were quantified by flow cytometry. Anti-IL-26 serum but not pre-immune serum partially neutralized IL-26 dependent effects compared to medium-treated VSV (Figure 4B).

The inhibition of the HCMV infection of IL-26-treated fibroblasts of about 50% was further quantified by flow cytometry in comparison to the control protein IL-2. The percentage of infected cells on d 5 post infection is shown (Figure 4C). Similarly to the IL-26-dependent enhanced VSV infection, the IL-26-dependent inhibition of HCMV-infection of fibroblasts was partially neutralized with the anti-IL-26 serum, in contrast to pre-immune serum (Figure 4D). The anti-IL-26 serum was not sufficient to fully block very high IL-26 concentrations (5 µg/ml or more). The numbers of HSV-1-infected primary fibroblasts on d 1 and 2 post infection were not relevantly altered by IL-26 in a similar experimental setup (Figure 4E). In summary, IL-26-dependent effects on VSV and HCMV infection of appropriate target cells can be neutralized with anti-IL-26 polyclonal serum in a dose-dependent manner.

### IL-26R-independence of IL-26-enhanced VSV infection

**Figure 5:**
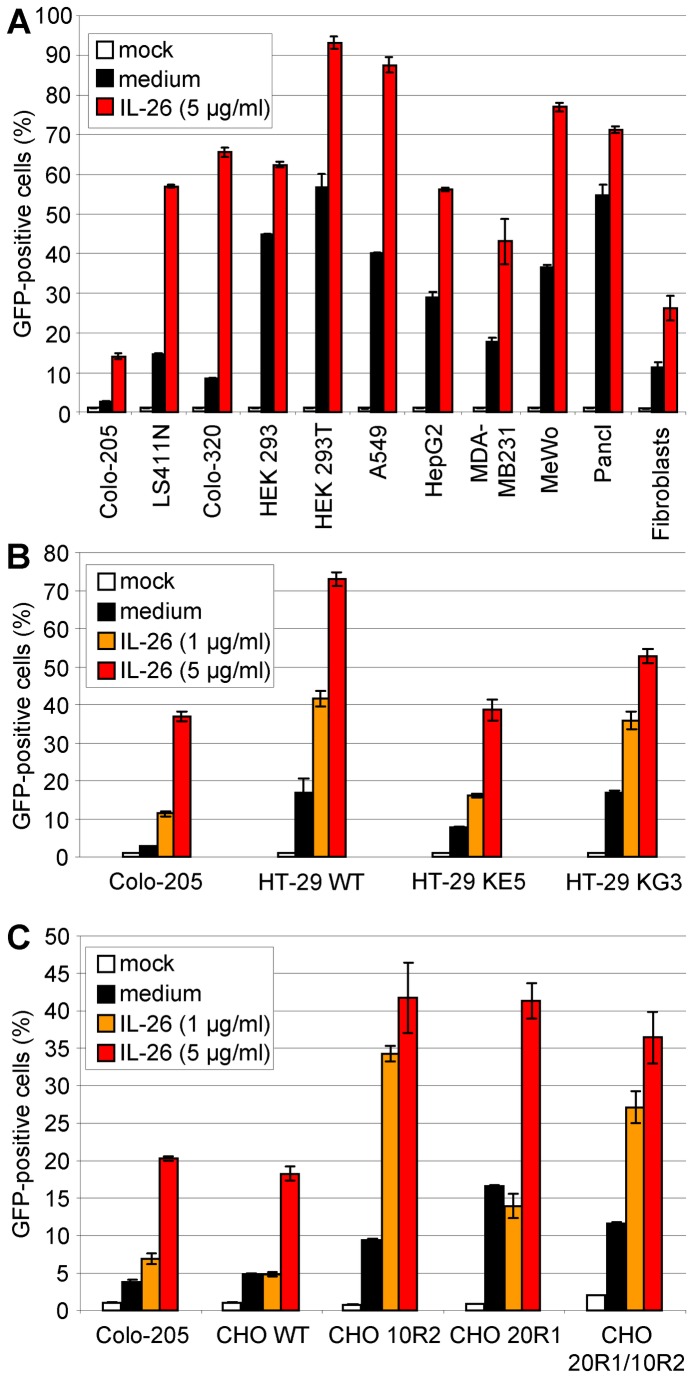
IL-26R-independence of IL-26-enhanced VSV infection. (A) Various cell lines with (Colo-205, LS411N) and without IL-26R (other cells) were pre-incubated with IL-26 (5 µg/ml) and subsequently infected with VSV (MOI = 0.01). After varying time points, cells were fixed with 2% PFA and GFP-positive cells were measured by flow cytometry. Mean results and standard deviation of a representative experiment are shown. (B) The IL-26 non-responsive cell line HT-29 wild type (WT) and the stably transfected IL-20R1 chain-expressing cloned derivates HT-29 KE5 and KG3 showed similar infection rates after pre-incubation with IL-26 (1 or 5 µg/ml) as Colo-205 cells as measured by flow cytometry after fixation 12 h post infection. Mean values with standard deviations of a representative experiment are shown. (C) Wild-type CHO (WT) cells which do not react on IL-26 and the IL-20R1-, IL-10R2-, or IL-20R1/IL-10R2-expressing derivates showed similar infection rates after pre-incubation with IL-26 (1 or 5 µg/ml) as Colo-205 cells as measured by flow cytometry after fixation 12 h post infection. Mean values with standard deviations of a representative experiment are shown.

Since it is known that IL-26 signals via its specific cytokine receptor, we investigated if the IL-26 effect on VSV infection is mediated by IL-26R consisting of IL-20R1 and IL-10R2 or by non-specific binding effects on the VSV particles or on target cells. We infected various carcinoma cell lines of different origins with (Colo-205, LS411N) or without IL-20R1 expression (all other cell lines tested [7,13,15], and additional RT-PCR data not shown for HEK293T, MDA-MB231, and primary fibroblasts) (Figure 5A). The accessory IL-10R2 chain is ubiquitously expressed and, therefore, not limiting for IL-26 responsiveness. Independently of the IL-20R1 expression status, all cell lines showed increased infection rates after IL-26 pre-incubation of target cells at variable extent as analyzed by flow cytometry (Figure 5A). Therefore, an IL-26R-independent mechanism seemed likely for the increased VSV infectivity.

**Figure 6:**
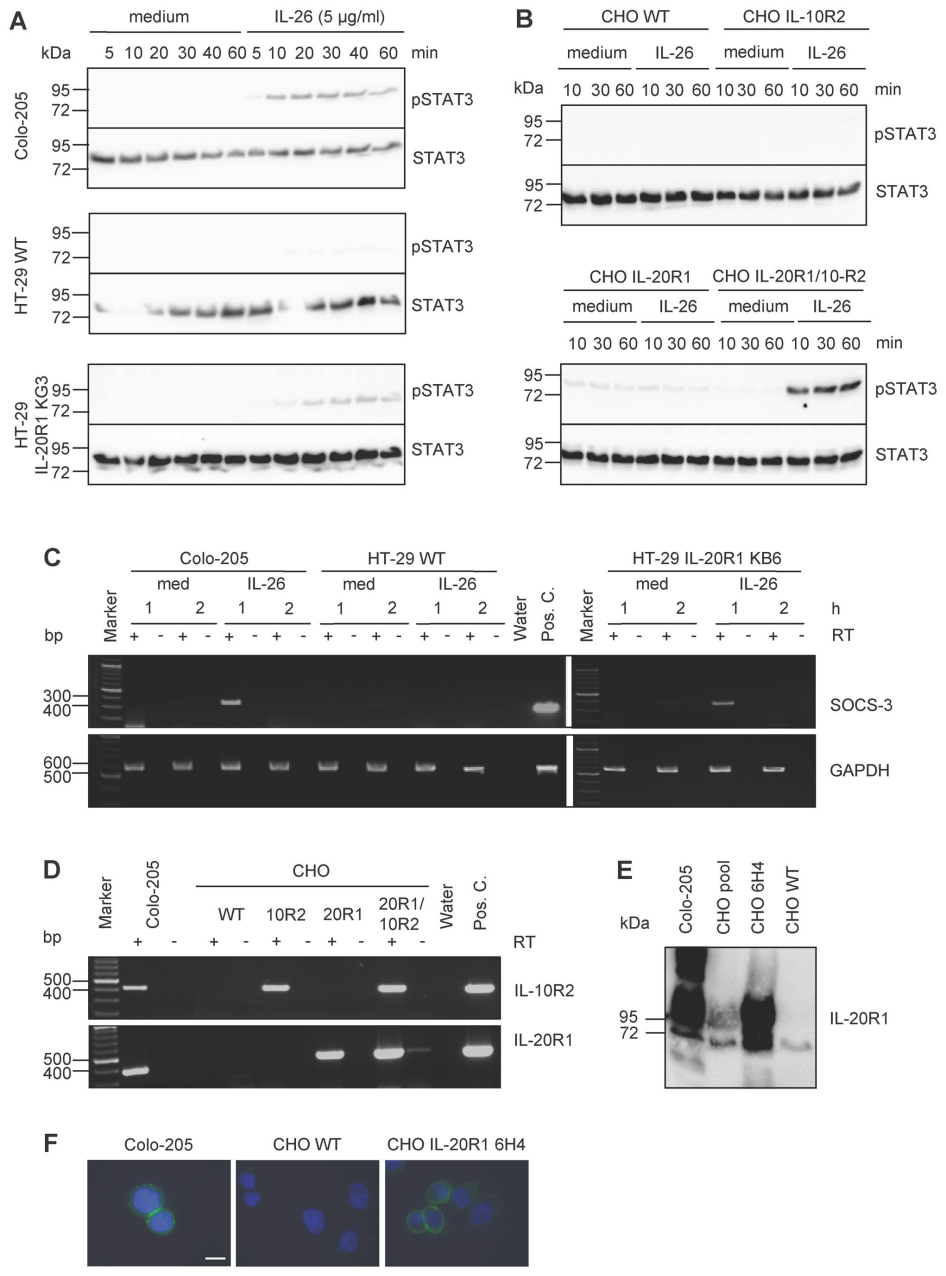
IL-26 induced STAT3 activation in IL-26R-reconstituted HT-29 and CHO cells. (A) IL-26 induced STAT3 activation (upper panels) in Colo-205 cells, HT-29 WT cells, and HT-29 IL-20R1 KG3 cells with STAT3 protein detection as loading control (lower panels). (B) IL-26 induced STAT3 activation (upper panels) in CHO cells expressing no IL-26R wild type (WT), IL-10R2, or IL-20R1 alone or both IL-20R1/IL-10R2 receptor chains and STAT3 protein detection (lower panels). (C) IL-26 induced *SOCS3* transcription in stably IL-20R1 expressing HT-29 cells relative to Colo-205 and wild type HT-29 cells. Total cellular RNA was analyzed 1 or 2 h after IL-26 stimulation by RT-PCR. *GAPDH* served as internal control. (D) IL-10R2 and IL-20R1 transcription after stable transfection of CHO cells, shown by RT-PCR. IL-20R1 protein expression in Colo-205, CHO WT cells, and the stably IL-20R1-transfected CHO clone 6H4 was shown by Western blot (E) and by immunofluorescence (F) with nuclear 4',6-diamidino-2-phenylindole stain (DAPI, scale bar = 20 µm).

In addition, IL-26-non responsive cells were stably transfected with IL-26R in order to generate responsiveness to IL-26-dependent signalling. This approach was preferred since transfection of Colo-205 cells is inefficient and precludes a knock-down strategy for the endogenous IL-26R chains. Therefore, VSV-susceptible, but IL-26 non-responsive cells were selected for further analysis and equipped with a functional IL-26R. The HT-29 colon carcinoma cell line used in our laboratory does not express an endogenous functional IL-26R and induces only minimal STAT3 phosphorylation after IL-26 stimulation (Figure 6A). The functional IL-26R was generated in HT-29 by stable transfection of IL-20R1 which led to IL-26-responsiveness via STAT3 phosphorylation (Figure 6A) and increased *SOCS3* transcription (Figure 6C). Additionally, IL-20R1 protein was detected by Western blot. HT-29 cells with or without functional IL-26R showed similar IL-26-inducible VSV-infection rates as Colo-205 cells and non-transfected parental HT-29 cells (Figure 5B), excluding an essential role of the IL-20R1 chain. This was confirmed by restoring both IL-26R chains in non-human CHO cells which showed the IL-26-mediated increased VSV infection similarly in parental cells and stably transfected derivates (Figure 5C). In contrast, only the transfected cells were able to respond with STAT3 activation and *SOCS3* transcription on IL-26 stimulation (Figure 6B). IL-20R1 expression in CHO cells was verified by RT-PCR (Figure 6D), cell surface flow cytometry, Western blot (Figure 6E), and fluorescence microscopy (Figure 6F), using IL-20R1 monoclonal antibodies produced in our laboratory.

### Virus particles as the probable target of the IL-26-dependent VSV enhancement

**Figure 7:**
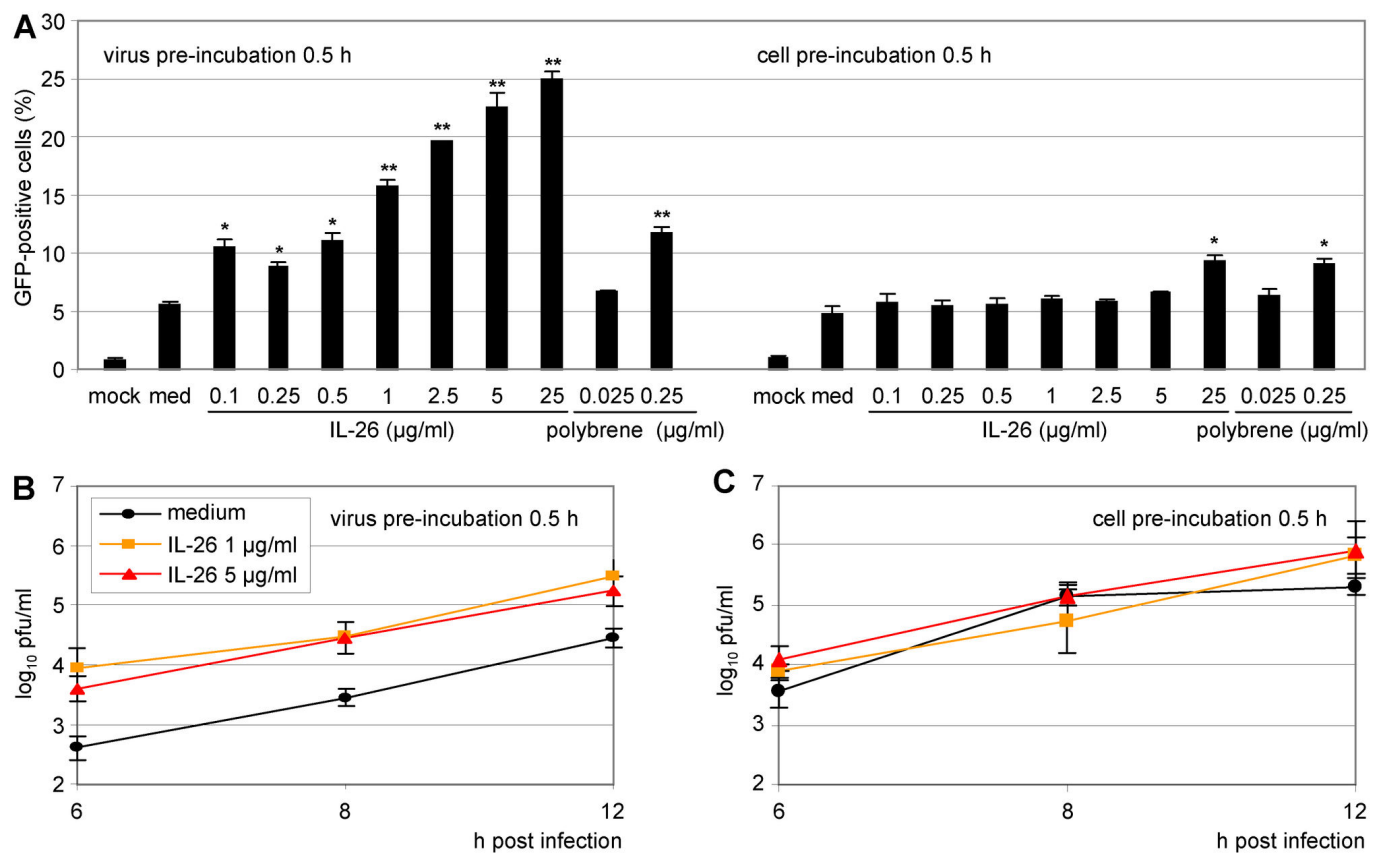
Strongest enhancement of VSV infection by pre-incubation of virus with IL-26. (A) Either VSV-GFP supernatant or Colo-205 target cells were pre-incubated for 0.5 h with IL-26 (0.1-25 µg/ml) or polybrene (0.025-0.25 µg/ml). Infected cells were fixed with PFA after 12 h and GFP-positive cells were measured by flow cytometry. Mean results with standard deviations are shown. (B) VSV supernatant or Colo-205 cells were pre-incubated with IL-26 (1 or 5 µg/ml) for 0.5 h. After depicted time points, supernatants were collected and titrated on Vero cells (log_10_ pfu/ml). The symbols * and ** indicate *P* values of <0.05 and <0.01, respectively, relative to medium (A), as determined by the Student’s t test.

In order to define the mechanism of the IL-26 effect on VSV infection more closely, different infection conditions were compared. Either the virus inocula or the permissive target cells were pre-incubated with IL-26. VSV or Colo-205 cells were pre-incubated with IL-26 (0.1-25 µg/ml) for 30 min and the infected cells were quantified by flow cytometry after 12 h (Figure 7A). The pre-incubation of virus with IL-26 was much more efficient in increasing VSV infection than the pre-incubation of the target cells. As positive control, the synthetic cationic polymer hexadimethrine bromide (polybrene) was included in the assay. The flow cytometry results were confirmed by the titration of infectious virions released into the supernatant after pre-incubation of target cells or VSV. The supernatants were collected at 6-12 h post infection and the titration was performed on Vero cells. If VSV particles in the supernatant were directly pre-treated with IL-26 (5 µg/ml), the yielding titer was approximately ten-fold higher than after mock-treatment or after cell pre-incubation of the target cells (Figure 7B, C). This indicates that IL-26-dependent effects on VSV infection are rather a result of the interaction between virions and the cytokine than of cell signalling events in the target cells.

**Figure 8:**
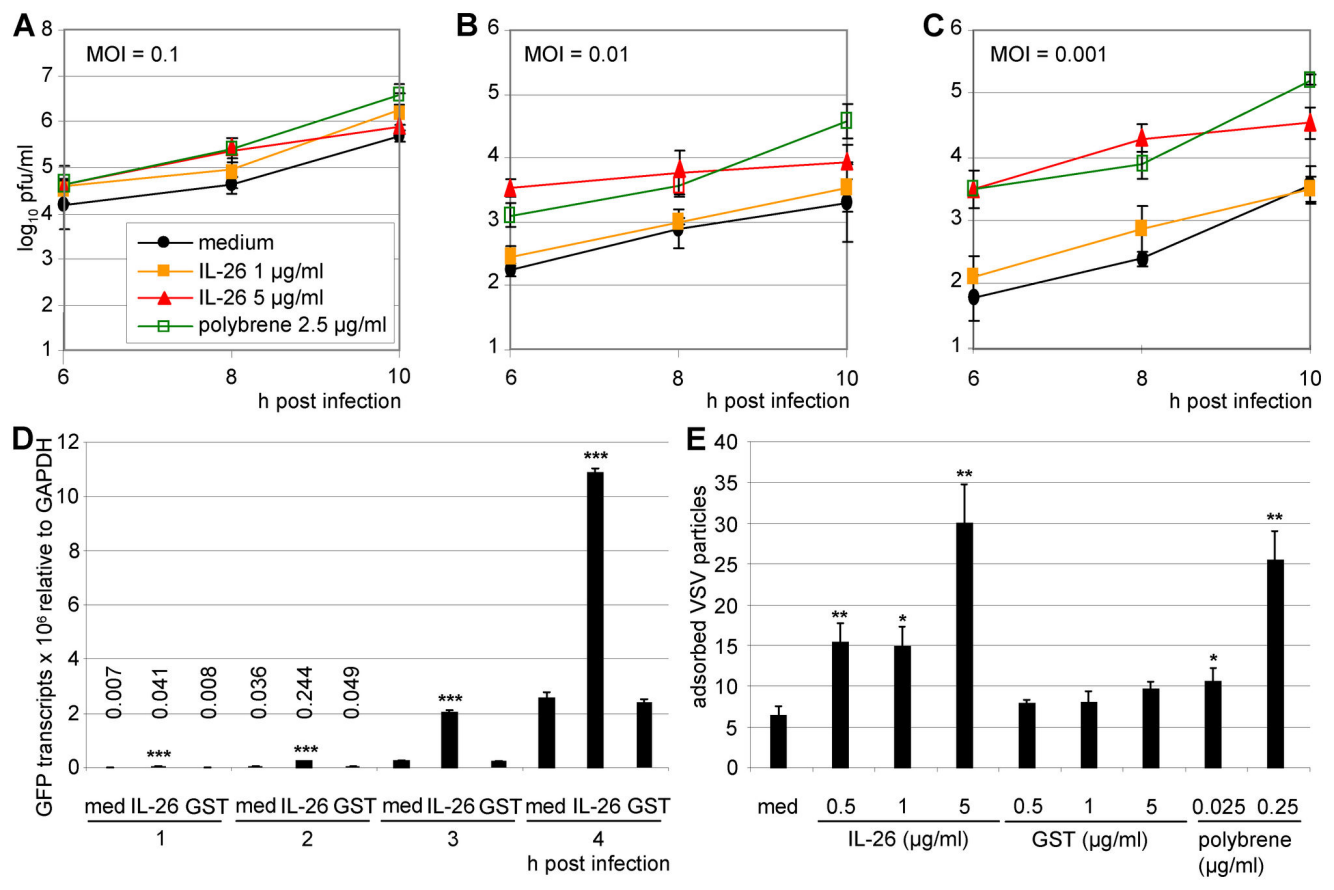
Strong enhancement effect of IL-26 at low VSV multiplicity, acting already during the adsorption step. (A–C) VSV-GFP was pre-incubated with IL-26 (1 or 5 µg/ml) or polybrene (2.5 µg/ml) and used for infection of Colo-205 cells at MOIs of 0.1, 0.01, and 0.001. Supernatants were collected after 6, 8, and 12 h and titrated on Vero cells (log_10_ pfu/ ml). (D) VSV-GFP was pre-incubated with IL-26 or GST (1 µg/ml) and used for infection of Colo-205 cells (MOI = 0.01). After 1-4 h, GFP transcription was analyzed by two-step quantitative RT-PCR relative to *GAPDH* reference expression. (E) VSV-GFP was pre-incubated with IL-26, GST (1 or 5 µg/ml), polybrene (0.25 or 0.025 µg/ml), or medium for 15 min. Adsorption to Colo-205 cells was done for 20 min on ice. Adsorbed viral genomes (GFP) were analyzed by two-step quantitative RT-PCR relative to cellular *GAPDH* reference expression. Mean results from triplicates with standard deviations of a representative experiment are shown. The *P* values for IL-26 (5 µg/ml, 8 h) were <0.01 relative to medium, for polybrene (8 h) <0.01, and for IL-26 (1 µg/ml, 10 h) < 0.05 (A), for IL-26 (5 µg/ml, 6 h) <0.001, and IL-26 (5 µg/ml, 8 h) < 0.05, and for polybrene (6 h) <0.001, polybrene (8 and 10 h) < 0.05 (B), and for IL-26 (5 µg/ml, 6 and 8 h) < 0.001 and IL-26 (10 h) < 0.01, and polybrene (6, 8 and 10 h) <0.001 (C). The symbols *, **, and *** (D, E) indicates *P* values of <0.05, <0.01, and <0.001 respectively, relative to medium, as determined by the Student’s t test.

The VSV MOI was crucial for the IL-26-dependent effects. Colo-205 cells were infected with pre-treated VSV with MOIs from 0.01–0.001. Supernatants were collected at 6, 8, and 10 h post infection and titrated on Vero cells (Figure 8A, B, C). The IL-26-dependent enhancement was optimal at low VSV MOI. This is consistent with the highest IL-26-dependent inhibition of HCMV infection at low MOI (data not shown).

The virus-encoded GFP transcription in Colo-205 after VSV infection was analyzed from 1–4 h post infection by quantitative RT-PCR in order to determine the very early time course of the IL-26-dependent effects on VSV replication. Virus-encoded GFP transcription was upregulated five- to eight-fold after IL-26 pre-treatment of Colo-205 cells as compared to medium or GST pre-incubated VSV. The values were normalized on *GAPDH* as house-keeping gene. This indicates that the number of infected target cells after IL-26-treatment of VSV particles is substantially higher in the very early infection phase, presumably by facilitated VSV attachment and entry into target cells (Figure 8D).

This hypothesis was further confirmed by the quantitative analysis of VSV binding to target cells. IL-26 enhanced VSV adsorption to Colo-205 cells after pre-incubation of VSV-supernatants with the cytokine (Figure 8E). The amount of the genomic VSV RNA was increased three- to six-fold compared to medium control depending on the IL-26 concentration. In contrast, GST pre-incubation did not alter VSV adsorption. Thus, the IL-26-dependent enhancement of VSV infection is observed already during the very first adsorption phase.

## Discussion

The cationic IL-10 family cytokine IL-26 is produced mainly by activated T cells and targets epithelial target cells for signal transduction via IL-26R. Here, we describe novel IL-26R-independent effects of the human cytokine IL-26 on virus infection. Whereas IL-26 induced a strong enhancement of VSV binding, infection, and replication, the HCMV-infection of IL-26-treated human fibroblasts was inhibited, and HSV-1 infection was not influenced. Thus, IL-26 differentially modulates the infection by different enveloped viruses.

IL-26 was originally discovered due to its over-expression in human T cells which are transformed by herpesvirus *Saimiri* [10]. Therefore, a role of IL-26 in virus infection seemed possible. This hypothesis was further supported by the antiviral function of other members of the IL-10 family, the IFN-λ proteins [43,44]. However, neither IL-26 nor IL-22 influenced the expression of typical IFN-regulated genes, such as *2’-5’-OAS*, *IFNB*, *MxA*, or *ISG15*, but both strongly induced the rapid expression of the counter-regulatory *SOCS3* gene, similarly to type I IFNs and IFN-λs ( [34,54,55], own unpublished observations, Figure 1D). The rapid and strong *SOCS3* induction could be involved in the IL-26 effect on virus infection by inhibiting the initial IFN response. Additionally, the gene for the transcription factor IRF1 was induced by IL-26 in Colo-205 cells. This may become relevant since single-stranded RNA viruses such as VSV and Sendai virus interact with post-translationally modified IRF-1 [56]. Also IL-26 induced a rapid posttranscriptional modification of the IRF-1 protein (data not shown), which might interfere with virus replication. *SOCS3*, *IRF-1*, and *STAT3* are induced by both IL-26 and IL-22 ( [19,34], own unpublished observations).

We analyzed the in vitro effects of IL-26 on virus infection and replication with recombinant viruses from three different species, the RNA rhabdovirus VSV, and the DNA herpesviruses HCMV and HSV-1. A major advantage of VSV and HSV-1 is their broad range of susceptible target cells. The three viruses expressed the GFP protein during infection which facilitated the detection and quantification of infected cells. As a first approach, we analyzed the number of Colo-205 cells infected with VSV in comparison to HSV-1 by fluorescence microscopy. Therefore, the viruses or Colo-205 target cells were pre-incubated with IL-26 and subsequently used for infection because Colo-205 cells are known to express the functional IL-26R. There was a strong increase of the number of VSV-infected cells after IL-26 pre-treatment of virus and slightly more infected cells by pre-incubation of Colo-205 cells. In contrast, the number of HSV-1-infected cells was not altered if virus or cells were pre-incubated. Since Colo-205 cells are not susceptible for HCMV infection, primary human fibroblasts were used for infection analysis. A moderate inhibition of HCMV infection with rather high IL-26 concentrations was observed, regardless of whether cells or virus were treated (Figure 1).

The IL-26-mediated enhancement of VSV infection, the inhibition of HCMV infection, and the unchanged HSV-1 infection were further confirmed and quantified by flow cytometry and by virus titration (Figure 2). Moreover, the direct measurement of fluorescence within the culture was used for the homogenous culture conditions of fibroblasts and Vero cells. Since Colo-205 cells tend to form heterogeneous monolayers and infection patterns, the microfluorimetry was not applicable for this cell type. The IL-26 effects on HCMV and HSV-1 infection and replication were confirmed and compared to the antiviral effects of GCV and ACV. The antiviral effect of IL-26 was in a similar range in comparison to that of GCV (Figure 3).

The predicted highly basic pI = 10.7 is the specific peculiarity of the IL-26 protein in comparison to all other members of the cytokine family [10]. Therefore, we compared the IL-26 effects to proteins with similar size, but without cationic properties. The recombinant His-tagged GST protein with a predicted pI = 6.7 and recombinant IL-2 (pI = 7.7) did not alter infection and replication of the three viruses, respectively. Although heparin prevents binding of IL-26 to its receptor on epithelial cells [15], it was not an appropriate control since it blocked VSV infection (data not shown). The specificity of IL-26-dependent enhancement of the VSV infection could be demonstrated with anti-IL-26 serum. Pre-treatment with the anti-IL-26 serum partially neutralized the enhancement of VSV infection and replication up to an IL-26 concentration of 1 µg/ml. The neutralization of the IL-26 inhibition of HCMV infection was only achieved partially probably since high IL-26 concentrations were used (Figure 4).

Since the cytokine IL-26 signals through its specific heterodimeric IL-26R, we analyzed the IL-26-dependent enhancement of VSV infection in various cell types with and without IL-20R1. Surprisingly, after IL-26 pre-treatment all cell types were more efficiently infected by VSV, as quantified by flow cytometry. This suggests that IL-26 affects VSV infection independently of IL-26R. Further, we stably expressed IL-20R1 in the colon carcinoma cell line HT-29 rendering this cell line responsive to IL-26. The numbers of VSV-infected HT-29 cells with or without IL-20R1 and with or without IL-26 pre-treatment were in a similar range. Similar results were obtained with stably transfected CHO cells, indicating that the IL-26 effect of VSV is independent of IL-20R1 (Figure 5). IL-26 shares its accessory receptor chain IL-10R2 with IL-10, IL-22, and the IFN-λ proteins. Since IL-10R2 is expressed by all the tested cell lines and CHO with or without IL-10R2 did not change their VSV infection pattern, a role for IL-10R2 in the observed IL-26-dependent VSV enhancement is largely excluded. Recent observations on the IL-26 effects on monocytes from rheumatoid arthritis patients also indicated independence from the classical IL-26R [16,40]. In addition, receptor-independent IL-26 effects were described on human B cells [57].

The pre-incubation of the VSV supernatant with recombinant IL-26 was much more efficient than the pre-incubation of the target cells and a considerably lower IL-26 concentration was required for enhancing virus infection (Figures 1, 6). Moreover, only low MOIs showed large differences between IL-26- or mock-treated VSV. Since already early time points after infection yielded obvious IL-26-effects, the first 4 h after VSV infection were examined more closely. Therefore, the transcription of virus-encoded GFP in VSV-infected cells was measured by RT-PCR. This indicated that IL-26-treated VSV was more successful in infection already after 2 h and argues in favour that VSV attachment and entry into target cells are facilitated by IL-26. This hypothesis was further confirmed by VSV binding analysis in which the IL-26 effect on VSV attachment was demonstrated on ice (Figure 8).

In contrast to VSV, the IL-26 effects on HCMV infection needed higher IL-26 concentrations and were comparable if the virions or the cells were pre-treated with the cytokine (data not shown). In the case of HSV-1, an influence of IL-26 was not observed, regardless of the IL-26 concentration and virus or cell pre-treatment (Figures 1-4).

The binding of virus particles to target cells depends on electrostatic interactions with the cell membrane. The polycation diethylaminoethyl dextran increased the binding of VSV to BHK cells about four-fold [58] and force spectroscopy experiments suggested that negatively charged phospholipids are required for VSV binding to membranes, showing an electrostatic interaction [59]. Hexadimethrine bromide (polybrene) is a synthetic cationic polymer that is used to enhance retrovirus infection by attenuating charge repulsion between the virion and the cellular target membrane [60,61]. In the case of human immunodeficiency virus, a cationic peptide fragment of the prostatic acidic phosphatase designated Semen-derived Enhancer of Virus Infection (SEVI) forms fibrils that drastically enhance infection [62]. The SEVI peptide has comparable polycationic properties to IL-26 with pI = 10.21. The positively charged amino acids Lys and Arg of SEVI seem to facilitate virion attachment and fusion to target cells presumably by neutralizing electrostatic repulsion between virion membranes [63]. This effect could be neutralized by polyanions such as heparin, dextran sulfate, oversulfated heparin, or oversulfated chondroitin sulfate by blocking the binding of SEVI to the virions [63]. Moreover, various cationic defensin peptides have shown antiviral activity [64,65]. In contrast to defensins and antimicrobial peptides, IL-26 did not show antibacterial activity (data not shown). Probably due to its positive charges, IL-26 sticks to cellular and possibly also viral surfaces thereby reaching high local protein concentrations. IL-26 cytokine effects were neutralized by negatively charged glycosaminoglycans [15]. Thus, IL-26 seems to interfere with the negatively charged surfaces of viruses and target cells and leads to differential effects on virus infection.

In summary, the T cellular cytokine IL-26 differentially modulates VSV, HCMV, and HSV-1 infection in vitro. Whereas VSV infection was strongly enhanced by IL-26, HCMV infection was inhibited, and HSV-1 infection was not influenced. The modulation may be explained by the cationic properties of the IL-26 protein. The enhanced VSV infection could result from the reduced repulsion between virus and target cell membranes and from increased infection efficiency, since the internalization of VSV mediated by clathrin-mediated endocytosis is both fast and inefficient [66]. Already initially, cells could be infected more efficiently and the differences between medium- or IL-26-treated viruses would be increased after several rounds of replication.
